# Conserved Linear Epitopes in the VP1 Protein of Foot‐and‐Mouth Disease Virus Serotype SAT2: Identification and Characterization

**DOI:** 10.1155/tbed/2217447

**Published:** 2026-04-22

**Authors:** Yu-qian Zhu, Xin-tai Shi, Alison Burman, Zheng-wang Shi, Tao Xi, Huan-cheng Liao, Jun-cong Luo, Jie Chen, Lin Wang, Ya-ge Xie, Qian-qian Yang, Hong Tian, Hai-xue Zheng, Donald P. King

**Affiliations:** ^1^ State Key Laboratory for Animal Disease Control and Prevention, College of Veterinary Medicine, Lanzhou Veterinary Research Institute, Lanzhou University, Chinese Academy of Agricultural Sciences, Lanzhou, 730046, China, lzu.edu.cn; ^2^ College of Veterinary Medicine, Gansu Agricultural University, Lanzhou, 730070, China, gsau.edu.cn; ^3^ School of Basic Medical Sciences, Gansu Medical College, Pingliang, 744000, Gansu, China; ^4^ FAO World Reference Laboratory for FMD, The Pirbright Institute, Pirbright, GU24 0NF, UK, pirbright.ac.uk

**Keywords:** diagnostic target, epitope conservation, foot-and-mouth disease virus, SAT2, VP1 linear epitope

## Abstract

Foot‐and‐mouth disease virus (FMDV) serotype Southern African Territories 2 (SAT2), historically endemic to sub‐Saharan Africa, has recently expanded its geographical range to the Middle East, posing a significant transboundary threat to global livestock industries. Characterized by high genetic diversity encompassing 14 distinct topotypes, SAT2 poses a challenge to reliable serological diagnosis and underscores the need for conserved, serotype‐specific antigenic targets. In this study, we aimed to identify such targets by mapping linear epitopes in the VP1 capsid protein using three monoclonal antibodies (mAbs; 3B2, 6F2, and 2F11) raised against SAT2 VP1. Epitopes were mapped through sequential truncation and alanine‑scanning mutagenesis, and their conservation was assessed across VP1 sequences from 53 SAT strains (35 SAT2, 13 SAT1, and 5 SAT3). The epitope recognized by mAb 3B2 was identified as ^204^RFDAPIGVE^212^. It displayed 88.9% sequence identity within SAT2 topotypes I, II, III, and VI, and 100% identity in all other topotypes, while showing minimal similarity with SAT1 and SAT3 VP1 proteins—confirming its serotype specificity. Through systematic alanine scanning, Asp206 was defined as a critical binding residue and was 100% conserved across all SAT2 topotypes, including recent outbreak strains. Structural analysis further indicated that this epitope is surface‑exposed and possesses high solvent accessibility. In contrast, the ^47^TSFVVDL^53^ epitope (recognized by mAb 6F2) was predominantly conserved only within SAT2 topotype VII, with limited conservation in other topotypes. The ^7^GAD^9^ core recognition sequence (targeted by mAb 2F11) showed variable conservation (66.7%–100%) among SAT strains. Collectively, the broad conservation across topotypes, combined with favorable structural features, positions the ^204^RFDAPIGVE^212^ epitope as a promising candidate for developing robust, SAT2‑specific serological diagnostics.

## 1. Introduction

Foot‐and‐mouth disease (FMD), caused by FMD virus (FMDV), is a highly contagious transboundary animal disease of global significance [1, 2].It is primarily characterized by vesicular lesions affecting cloven‐hoofed livestock and can result in severe production losses, particularly within intensively managed animal populations [3, 4]. Owing to its extreme transmissibility and the expanding scale of international trade in animals and animal products, FMD continues to pose a persistent biosecurity threat to countries that are free from the disease [5]. Moreover, it remains one of the most economically devastating livestock diseases in many developing countries and regions worldwide [6]. In response to these challenges, the World Organization for Animal Health (WOAH) has emphasized the importance of global coordination and regionally integrated control strategies to mitigate the impact of FMD [7].

Among the seven serotypes of FMDV (O, A, C, Asia1, and Southern African Territories (SAT; SAT1, SAT2, and SAT3), the SAT serotypes display the greatest genetic and antigenic variability [8, 9]. Notably, FMDV serotype SAT2, which was historically endemic to sub‐Saharan Africa, has emerged as a significant transboundary threat beyond its traditional endemic range. Recent incursions into the Middle East (e.g., Iraq, Jordan, and Türkiye) and the potential for further spread into East Asia [10–13] pose a direct threat to Chinaʼs swine industry—one of the largest livestock production systems worldwide, particularly through trade and animal movement pathways. The existence of 14 genetically distinct SAT2 topotypes, which exhibit minimal immunological cross‐protection, complicates control efforts in endemic regions and poses biosecurity challenges to disease‐free countries [14]. This extensive heterogeneity complicates vaccine strain selection and undermines effective disease control in endemic regions, while simultaneously increasing the biosecurity risks faced by previously FMD‐free countries. Of particular concern, the most recent outbreak of FMDV serotype SAT2 reported in Algeria in 2024 represented the first documented detection of this serotype in the Maghreb region of North Africa and was attributed to topotype V [15]. Multiple studies have identified livestock markets as key transmission hubs, underscoring the urgent need for robust cross‐regional surveillance and biosecurity measures [16, 17]. Despite the growing epidemiological threat posed by SAT2, effective surveillance remains severely constrained by the limited availability of serological diagnostic tools that have been specifically validated for this serotype. Consequently, the identification of conserved, serotype‐specific antigenic determinants within SAT2 is of paramount importance for the development of improved diagnostic assays and next‐generation vaccines.

The viral capsid protein VP1 has been a primary focus of such efforts. As a major structural component of the FMDV capsid, VP1 contains multiple key antigenic sites, including the highly variable G–H loop, which represents a principal target of the host humoral immune response and harbors immunodominant epitopes essential for antibody recognition [18, 19]. Previous studies have described several putative linear epitopes within the VP1 protein of SAT2, including regions spanning amino acid (aa) residues 42–61, 138−160, and 194–204 [20], as well as discrete antigenic sites located at positions 147–149, 156, and 158 [21].

Current advances in SAT2 diagnostics generally follow two complementary strategies. Molecular approaches, such as topotype‐specific real‐time RT‐PCR assays, have improved early detection and molecular epidemiological tracing; however, their performance is challenged by the extensive genetic variability among the 14 SAT2 topotypes [22]. In contrast, serological assays, including liquid‐phase blocking ELISA (LPBE), remain the cornerstone for antibody detection. Nevertheless, their diagnostic reliability is highly dependent on the antigenic concordance between assay antigens and circulating field strains, highlighting the critical need for conserved and serotype‐representative antigenic targets [23]. Although computational prediction and structural analyses have proposed several candidate antigenic regions, experimental identification and validation of linear epitopes that are both highly conserved across all SAT2 topotypes and suitable for standardized serological platforms (e.g., peptide‐based ELISA) remain limited.

Given the substantial heterogeneity of SAT2 and current diagnostic limitations, we postulated that highly conserved linear epitopes are present within the VP1 protein across all 14 SAT2 topotypes. Such epitopes would provide strain‐independent antigenic targets suitable for standardized serological platforms. Accordingly, this study systematically mapped and experimentally characterized linear epitopes within the SAT2 VP1, aiming to identify conserved antigenic determinants to facilitate the development of specific, broadly applicable diagnostic assays for SAT2 infection.

## 2. Materials and Methods

### 2.1. Cells, Strains, and Plasmids

Hybridoma cell lines were cultured in Roswell Park Memorial Institute (RPMI) 1640 medium (Gibco, USA) supplemented with 10% fetal bovine serum (FBS; Gibco, USA) and 1% penicillin–streptomycin (Solarbio Biotech, China) at 37°C in a humidified 5% carbon dioxide atmosphere. Hybridoma cell lines (3B2, 6F2, and 2F11) were preserved at the Lanzhou Veterinary Research Institute, Chinese Academy of Agricultural Sciences. SAT2‐VP1 truncated fragments were synthesized by gene synthesis (Genecreate.cn) and cloned into the pMAL‐c2x vector.

### 2.2. Preparation of Monoclonal Antibodies

The hybridoma cell lines (3B2, 6F2, and 2F11; [24]) were revived and cultured. The studies employed ascitic fluids for the three mAbs prepared according to the Experimental Animal Ethics Committee of the Lanzhou Veterinary Research Institute, Chinese Academy of Agricultural Sciences (LVRIAEC‐2023‐054).

### 2.3. Epitope Identification

To map the epitopes recognized by the three mAbs within the VP1 protein, VP1 was truncated into a series of overlapping fragments and cloned into the pMAL‐c2x vector. The recombinant plasmids were transformed into competent *E. coli* BL21 (DE3) cells. Transformed cells were cultured in Luria–Bertani broth supplemented with ampicillin (100 μg/mL) at 37°C with shaking at 220 rpm. When the optical density at 600 nm (OD_600_) reached 0.6–0.8, isopropyl β‐D‐1‐thiogalactopyranoside was added at a final concentration of 0.1 mM to induce protein expression for 6 h. The proteins were purified using an MBP Trap column. Sodium dodecyl sulfate‐polyacrylamide gel electrophoresis (SDS‐PAGE) was used to analyze protein expression and purity. Subsequently, the proteins were subjected to western blotting. Following SDS‐PAGE, the proteins were transferred onto nitrocellulose (NC) membranes using a standard wet transfer method. NC membranes were blocked with 5% (w/v) skim milk in Tris‐buffered saline containing 0.1% Tween‐20 (TBST) at room temperature (RT) for 2 h. The membranes were then incubated with the respective mAbs (diluted 1:5000) overnight at 4°C. After washing thrice with TBST (10 min per wash), the membranes were incubated with HRP‐labeled Goat anti‐mouse IgG (1:10,000; Abcam, Cambridge, MA, USA) at RT for 1 h. The membranes were then washed thrice with TBST, and protein bands were detected using an enhanced chemiluminescence substrate.

### 2.4. Homology Analysis

To assess the conservation of the identified epitopes among SAT serotypes, this study retrieved the VP1 amino acid sequences of 53 representative SAT strains (including 35 SAT2, 13 SAT1, and 5 SAT3 isolates) from the GenBank database. BioEdit software was used to align these sequences for analyzing the conservation characteristics of the epitopes. Additionally, based on the G–H loop region of SAT2 reported in Reference [25], this region was aligned with the target VP1 sequences to evaluate the conservation of the G–H loop. Detailed information of all strains is provided in Table 1.

**Table 1:** VP1 reference sequences of the 53 SAT strains analyzed.

Year	Area	Strain	Topotype	GenBank accession number
2019	Zambia	SAT2/ZAM/12/2019	Ⅰ	MZ486077.1
2020	Malawi	SAT2/MAL02/20	Ⅰ	UGN74726.1
1983	Zimbabwe	SAT2/ZIM/7/83	Ⅱ	DQ009726. 1
1998	Botswana	SAT2/BOT/P3/98	Ⅲ	KY091305. 1
1990	Ethiopia	SAT2/ETH/1/90	Ⅳ	AY343935. 1
1990	Ghana	SAT2/GHA/2/90	Ⅴ	AF479415. 1
1979	Gambia	SAT2/GAM/8/79	Ⅵ	AF479410. 1
1998	Eritrea	SAT2/ERI/12/98	Ⅶ	AF367126. 1
2000	South Africa	SAT2/SAU/6/00	Ⅶ	AF367135. 1
2014	Egypt	SAT2/EGY/24/2014	Ⅶ	KY825720. 1
2015	Ethiopia	SAT2/ETH/16/2015	Ⅶ	MT602090. 1
2018	Egypt	SAT2/EGY/Ismailia/2018	Ⅶ	MZ090097. 1
2018	Ethiopia	SAT2/ETH/11/2018	Ⅶ	MT602091. 1
2014	Nigeria	SAT2/NIG/1/14	Ⅶ	MN103523. 1
2018	Ghana	SAT2/GHA/Tul/5/2018	Ⅶ	LC456875. 1
2018	Ghana	SAT2/GHA/Tul/2/2018	Ⅶ	LC456874. 1
2012	Libya	SAT2/LIB/39/2012	Ⅶ	AFU55195.1
2017	Sudan	SAT2/SUD/7/2017	Ⅶ	QHI00030.1
2019	Central African Republic	SAT2/CAR/FMFP3/13/2019	Ⅶ	OR425072.1
2020	Nigeria	SAT2/NIG/91/2020	Ⅶ	XXP57879.1
2000	Rwanda	SAT2/RWA/1/00	Ⅷ	AF367134. 1
1957	Kenya	SAT2/KEN/3/57	Ⅸ	AJ251473. 1
1984	Kenya	SAT2/KEN/2/84	Ⅸ	AY343941. 1
1998	Uganda	SAT2/UGA/19/98	Ⅹ	AY343969. 1
1974	Angola	SAT2/ANG/4/74	XI	AF479417. 1
1975	Uganda	SAT2/UGA/51/75	XII	AY343963. 1
1977	Sudan	SAT2/SUD/6/77	XIII	AY343939. 1
2007	Ethiopia	SAT2/ETH/2/2007	XIII	FJ798161. 1
1991	Ethiopia	SAT2/ETH/2/91	XIV	AY343938. 1
2022	Ethiopia	SAT2/ETH/2/2022	XIV	WKE35517.1
2023	Jordan	SAT2/JOR/11/2023	XIV	WUR05443.1
2023	Oman	SAT2/OMN/23Z001438/2023	XIV	XQU63485.1
2023	Ethiopia	SAT2/ETH/1/2023	XIV	XQZ02670.1
2023	Jordan	SAT2/JOR/20/2023	XIV	XMD96899.1
2023	Turkey	SAT2/TUR/8/2023	XIV	XMD96906.1
2023	Iraq	SAT2/IRQ/3/2023	XIV	XMD96892.1
2003	Zimbabwe	SAT1/ZIM/23/2003	Ⅰ	KF219690.1
1970	Pirbright, WRL	SAT1/20 RV 11/37	Ⅱ	AY593839.1
1970	Botswana	SAT1/1 BEC	Ⅲ	AY593838.1
1970	Uganda	SAT1/UGA‐BUFF/21/70	Ⅳ	KF219682.1
1975	Nigeria	SAT1/NIG11/75	Ⅴ	AF431711.1
1976	Sudan	SAT1/SUD/3/76	Ⅵ	AY441996.1
1974	Uganda	SAT1/UGA/13/74	Ⅶ	AY442010.1
1997	Uganda	SAT1/UGA/1/97	Ⅷ	AY442012.1
2007	Ethiopia	SAT1/ETH/3/2007	Ⅸ	FJ798154.1
2015	Nigeria	SAT1/NIG/1/2015	X	KX822796.1
1972	Czechoslovakia	SAT 1/TCH/1/72	XI	MG972459.1
1974	Angola	SAT 1/ANG/9/74	XII	MG972460.1
2010	Mozambique	SAT 1/MOZ/P13/2010 B16	XIII	KF219691.1
1990	South Africa	SAT 3/KNP 10/90/3	Ⅰ	AF286347.1
1965	Botswana	SAT 3/4 BEC 1/65	Ⅱ	AY593853.1
1991	Zimbabwe	SAT3/ZIM/05/91	Ⅲ	MK415745.1
1996	Zambia	SAT 3/ZAM/04/96/3	Ⅳ	DQ009741.1
2013	Uganda	SAT 3/UGA/1/13	Ⅴ	KJ820999.1

### 2.5. Structural Accessibility Analysis

To evaluate the spatial accessibility of the identified linear epitopes, solvent‐accessible surface area (SASA) analysis was performed using the PyMOL Molecular Graphics System (v3.1.6). Based on the FMDV VP1 crystal structure, the VP1 monomer was extracted. Atomic selection sets corresponding to each epitope were created, and their total SASA was calculated using PyMOL’s get_area command, reflecting potential antibody accessibility under physiological conditions.

### 2.6. Statistical Analysis

All experiments were conducted in triplicate. Data are presented as mean ± standard deviation (SD). Statistical comparisons between two groups were performed using unpaired *t*‐tests in GraphPad Prism (Version 8.0). Differences were considered statistically significant at *p* < 0.05.

## 3. Results

### 3.1. Identification of Monoclonal Antibodies

SDS‐PAGE analysis confirmed the successful purification of mAbs 3B2, 6F2, and 2F11 (Figure 1A). Indirect ELISA revealed high titers (1:256,000; defined as a P/N ratio >2.1) for mAbs 3B2, 6F2, and 2F11 (Figure 1B). Specificity testing confirmed that mAb 3B2 reacted exclusively with SAT2 viruses and demonstrated no cross‐reactivity with representative strains of FMDV serotypes O, A, C, Asia1, SAT1, or SAT3 (Figure 1C, Table 2).

Figure 1Purification and specificity of mAbs against SAT2 VP1. (A) SDS‐PAGE (12% gel) of the purified mAbs 3B2, 6F2, and 2F11 under reducing conditions. Lane M: protein marker (kDa indicated); lanes 1–3: purified mAbs. (B) Titers of mAbs measured using indirect ELISA (1:256,000 dilution, cut‐off P/N > 2.1). (C) Serotype specificity of mAb 3B2.

**Figure:**
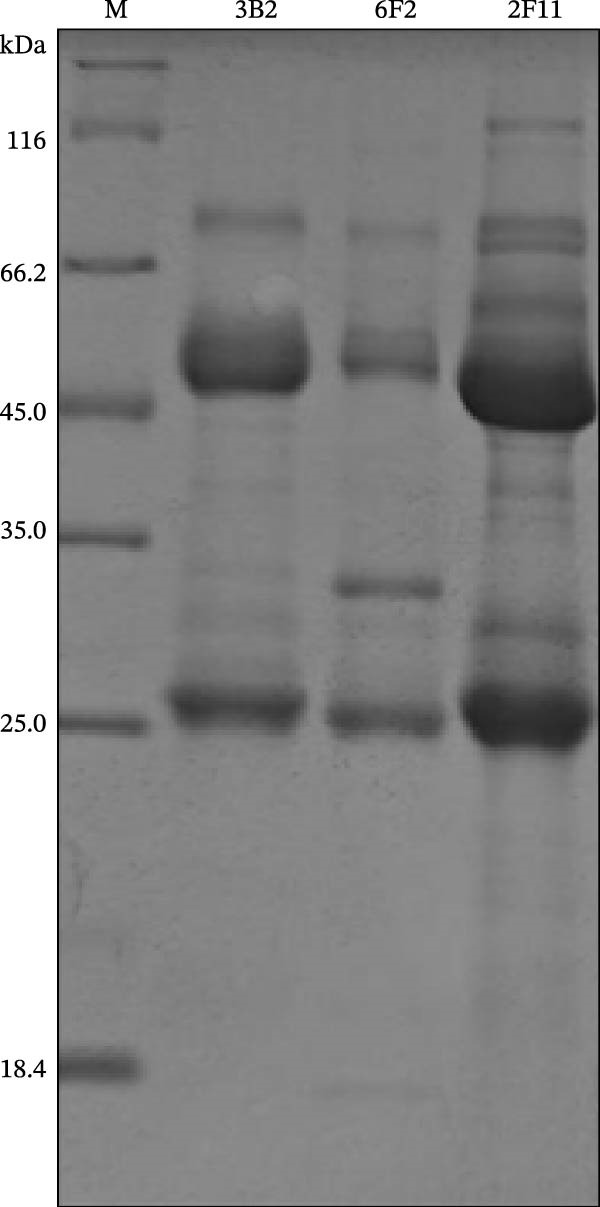
(A)

**Figure:**
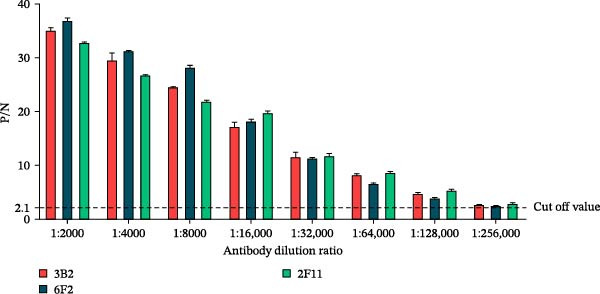
(B)

**Figure:**
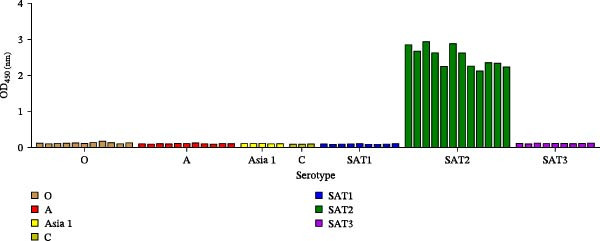
(C)

**Table 2:** The strain‐specific reactivity of mAb 3B2.

Serotype	Strain	Serotype	Strain	Serotype	Strain
O	EGY 18/2016	A	ZAM1/2015	Asia1	AFG1/2001
O	BAN 5/2009	A	EGY1/1972	Asia1	IRN 26/2015
O	KUW 3/1997	A	ETH 12/2009	Asia1	IND 18/1980
O	OMN 7/2001	A	IRN1/2016	Asia1	IRN 10/2004
O	NEP 17/2016	A	CAM5/2015	Asia1	NKR 2/2007
O	PAT 6/2015	A	SAU 15/2016	C	KENA/2004
O	MAY 3/2014	A	IRN1/2005	C	NEP 35/1996
O	MYA 5/2015	A	IRN 78/2009	C	PHI 3/1994
O	NIG 3/2014	A	MYA3/2015	SAT2	MOZ 3/2015
O	HKN 1/2015	A	PAK 56/2015	SAT2	ZIM 25/2015
O	TAN 4/2014	A	APG 6/2007	SAT2	BOT 3/2015
SAT1	KEN4/2013	SAT3	KNP 48/1991(Buffalo)	SAT2	ZAM 2/2015
SAT1	ZIM 14/2015	SAT3	RHO 5/1975	SAT2	UGA 9/1995
SAT1	NMB 1/2015	SAT3	SAR1/2006	SAT2	GHA 8/1991
SAT1	UGA 7/1999	SAT3	BOT P10/2010 (Buffalo)	SAT2	OMN 3/2015
SAT1	ETH 3/2007	SAT3	ZAM 3/2015	SAT2	EGA 44/2012
SAT1	MOZ 1/1975	SAT3	ZIM2/1984	SAT2	MAU 1/2014
SAT1	NIG 1/1976	SAT3	ZIM P25/1991 (UR‐7 Buffalo)	SAT2	UGA 2/2002
SAT1	NIG 3/1980	SAT3	ZAM P2/1996 (MUL‐4)	SAT2	UGA 3/1976
SAT1	UGA 47/1971	SAT3	UGA 10/1997	SAT2	SUD 6/1977

### 3.2. Epitope Mapping

To determine the precise epitopes recognized by the three mAbs, VP1 was truncated into five overlapping fragments (A1–A5) with 36‐aa overlaps (Figure 2A): A1 (1–72 aa), A2 (36–108 aa), A3 (72–144 aa), A4 (108–180 aa), and A5 (144–216 aa). Western blotting revealed that mAb 2F11 recognized A1 and 6F2 bound to both A1 and A2, whereas 3B2 specifically recognized the C‐terminal segment A5. Notably, none of the three mAbs reacted with the A3 or A4 region (Figure 2B).

Figure 2Epitope mapping of SAT2 VP1 using truncated fragments. (A) Schematic representation of the VP1 truncation strategy. Five overlapping fragments (A1: 1–72 aa, A2: 36–108 aa, A3: 72–144 aa, A4: 108–180 aa, and A5: 144–216 aa) were cloned into pMAL‐c2x for MBP‐tagged expression. (B) Western blotting of fragments A1–A5 probed with mAbs 3B2, 6F2, and 2F11 (1:5000 dilution). The reactive fragments are marked in red.

**Figure:**
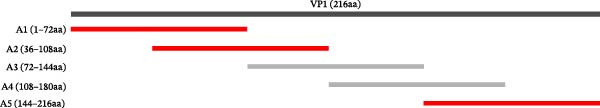
(A)

**Figure:**
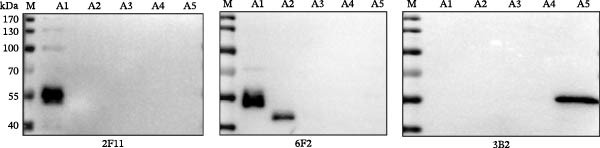
(B)

Based on the preliminary results, the epitope recognized by mAb 3B2 was initially subdivided into three overlapping fragments: B1 (181–203 aa), B2 (185–208 aa), and B3 (193–216 aa) (Figure 3A). mAb 3B2 specifically recognized the B3 fragment but not B2 (Figure 3B), indicating that the key epitopes lie within the C‑terminal region unique to B3 (residues 209–216). Thereby restricting the minimum epitope region to residues 209–216. To precisely map the critical residues, the region of interest was extended upstream by six amino acids (from residues 203–216) to ensure accurate identification. Systematic triple‐alanine‐scanning mutagenesis was performed across the extended region (residues 203–216), yielding 12 overlapping peptide mutants for detailed functional analyses (Figure 3C). Western blotting revealed that mutations within residues 204–212 (mutants C2–C10) abolished mAb 3B2 binding, whereas mutations outside this range (C1, C11, and C12) had no effect (Figure 3D). These results defined the minimal linear epitope as ^204^RFDAPIGVE^212^.

Figure 3Fine mapping of mAb 3B2 epitope using alanine‐scanning mutagenesis. (A) Truncation strategy for VP1 residues 181–216 aa. (B) Western blotting of fragments B1–B3 probed with mAb 3B2. (C) Design of 12 alanine mutants (C1–C12) with triple‐alanine substitutions at 203–216 aa, the red area indicates a mutation. (D) Western blotting validation of mutants. (E) Schematic diagram of single amino acid site mutation. (F) Key residues were validated by Western blotting, with quantitative densitometric analysis performed. Data are presented as mean ± standard deviation (SD), and error bars represent the SD of three independent experiments. Statistical significance was defined as follows: ns, no significance;  ^∗^ *p* < 0.05;  ^∗∗^ *p* < 0.01;  ^∗∗∗^ *p* < 0.001;  ^∗∗∗∗^ *p* < 0.0001. (G) Sequence alignment highlighting conservation of the core Asp206 residue.

**Figure:**
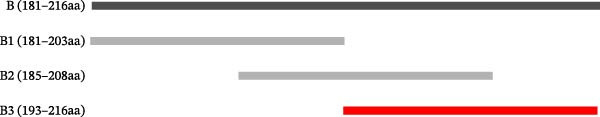
(A)

**Figure:**
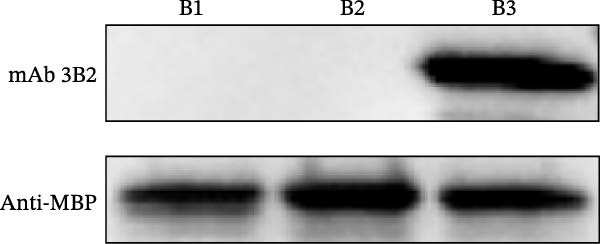
(B)

**Figure:**
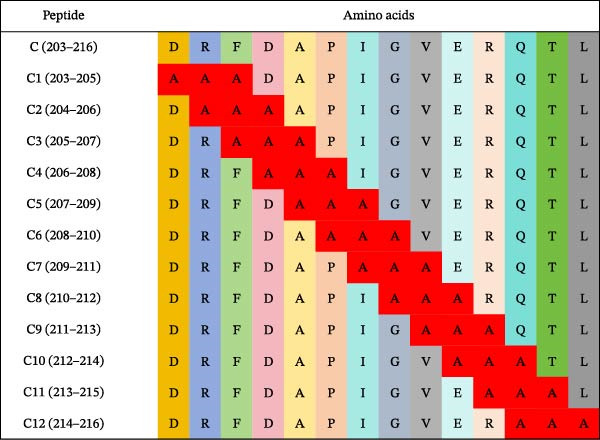
(C)

**Figure:**
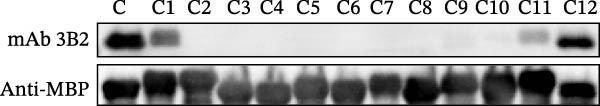
(D)

**Figure:**
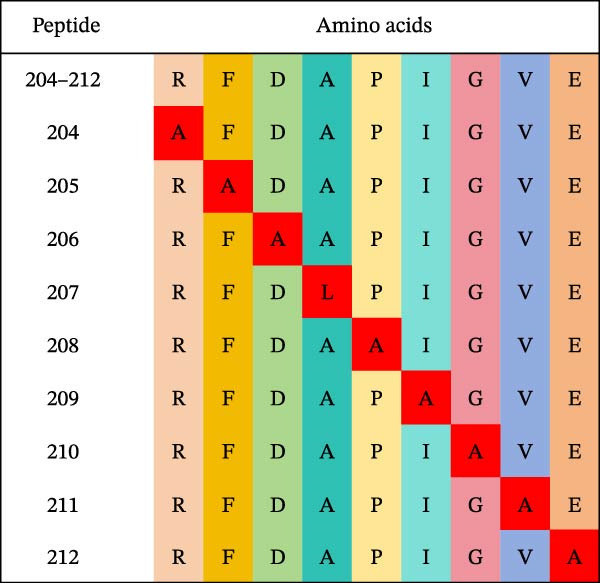
(E)

**Figure:**
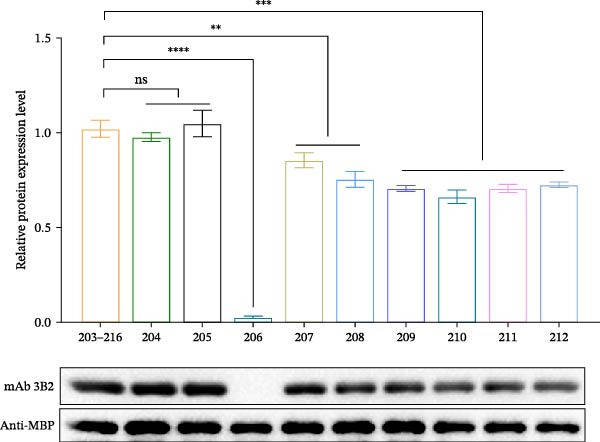
(F)

**Figure:**
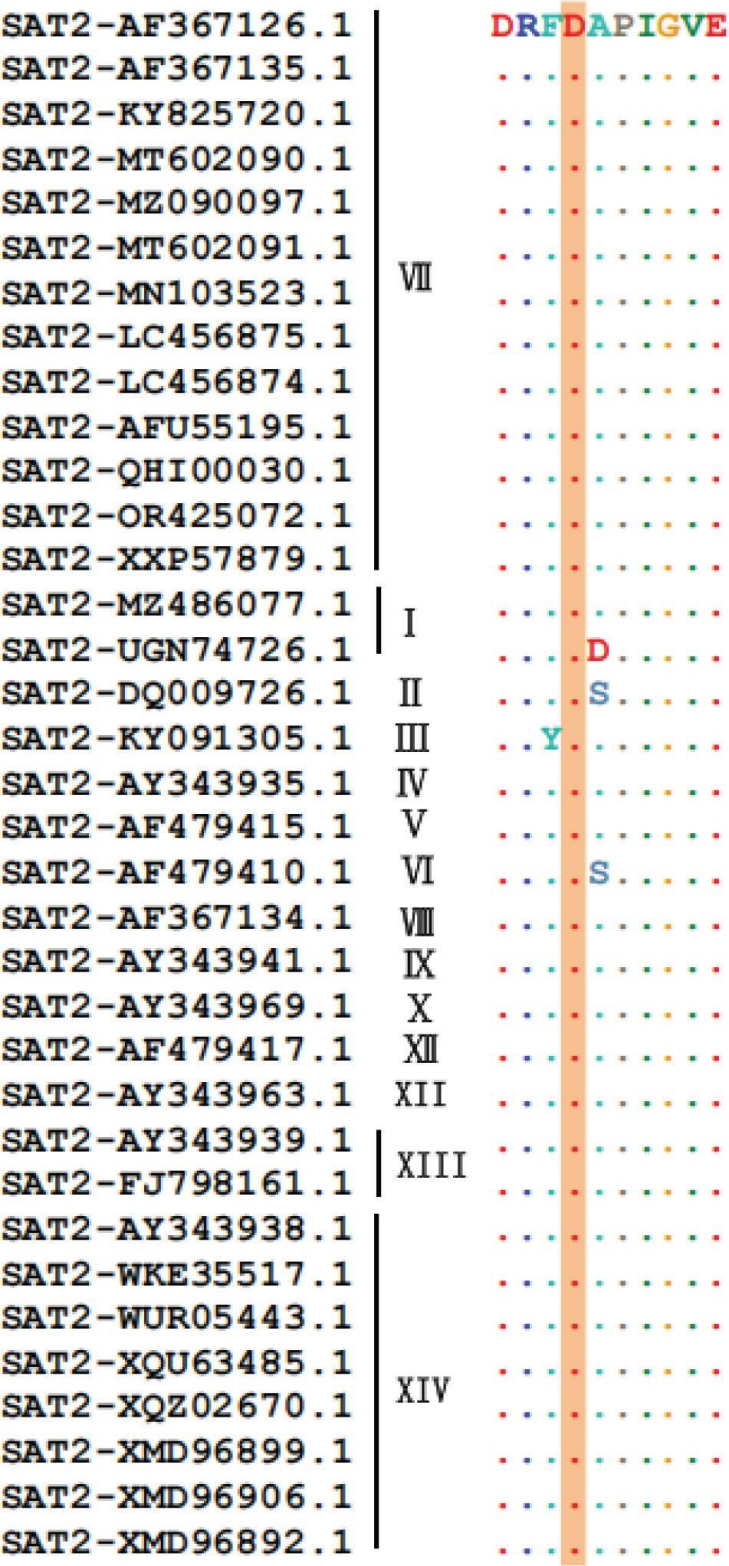
(G)

To characterize key residues within RFDAPIGVE, single‐alanine‐scanning mutagenesis was performed on each of the nine residues (Figure 3E). Western blotting analysis revealed that substitution at Asp206 completely abrogated mAb 3B2 binding, identifying this residue as critical for antibody recognition (Figure 3F). Quantitative analysis of band intensities further supported this conclusion (Figure 3F). Importantly, Asp206 was 100% conserved across all 14 SAT2 topotypes (Figure 3G).

Identification of 6F2 precise epitopes: We subdivided the epitopes (36–72 aa) identified by 6F2 into D1 (36–59 aa), D2 (41–64 aa), and D3 (49–72 aa) (Figure 4A). Western blotting results revealed that mAb 6F2 recognizes both D1 and D2 simultaneously (Figure 4B), indicating that the epitope lies between residues 41 and 59. To further refine the epitope recognized by mAb 6F2, we subdivided 41–59 aa into E1 (41–49 aa), E2 (46–54 aa), and E3 (51–59 aa) (Figure 4C). Western blotting analysis confirmed that mAb 6F2 recognized all three subfragments (Figure 4D), indicating that the epitope core lies within residues 46–54. To verify whether flanking residues (41–46 and 54–59 aa) contribute to epitope recognition, triple‐alanine‐scanning mutagenesis was performed on these regions to generate mutants F1–F4 (Figure 4E). Western blotting analysis showed that mAb 6F2 retained binding to all four flanking mutants (Figure 4F), demonstrating that these flanking residues are not essential for antibody recognition. Collectively, these data define the minimal linear epitope recognized by mAb 6F2 as ^47^TSFVVDL^53^.

Figure 4Identification of mAb 6F2 epitope in VP1. (A) Truncation strategy for VP1 36–72 aa. (B) Western blotting of D1–D3 fragments probed with mAb 6F2. (C) Further subdivision of the epitope region (41–59 aa) into E1–E3 fragments. (D) Western blotting of E1–E3. (E) Alanine mutants (F1–F4) of the flanking (41–46 aa, 54–59 aa), the red area indicates a mutation. (F) Western blotting of mutants. (G) Schematic of single‐alanine substitutions within TSFVVDL. (H) Key residues were validated by Western blotting, with quantitative densitometric analysis performed. Data are presented as mean ± standard deviation (SD), and error bars represent the SD of three independent experiments. Statistical significance was defined as follows: ns, no significance;  ^∗^ *p* < 0.05;  ^∗∗^ *p* < 0.01;  ^∗∗∗^ *p* < 0.001;  ^∗∗∗∗^ *p* < 0.0001.

**Figure:**
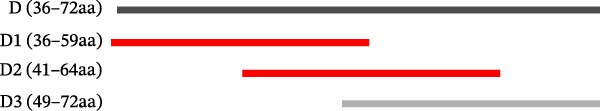
(A)

**Figure:**
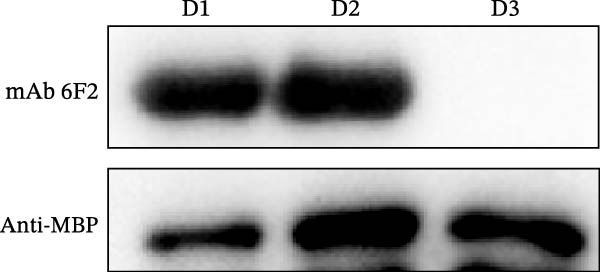
(B)

**Figure:**
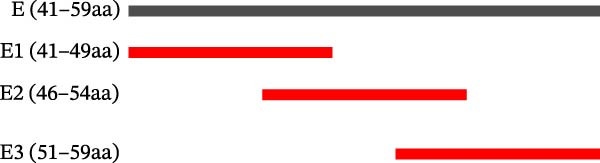
(C)

**Figure:**
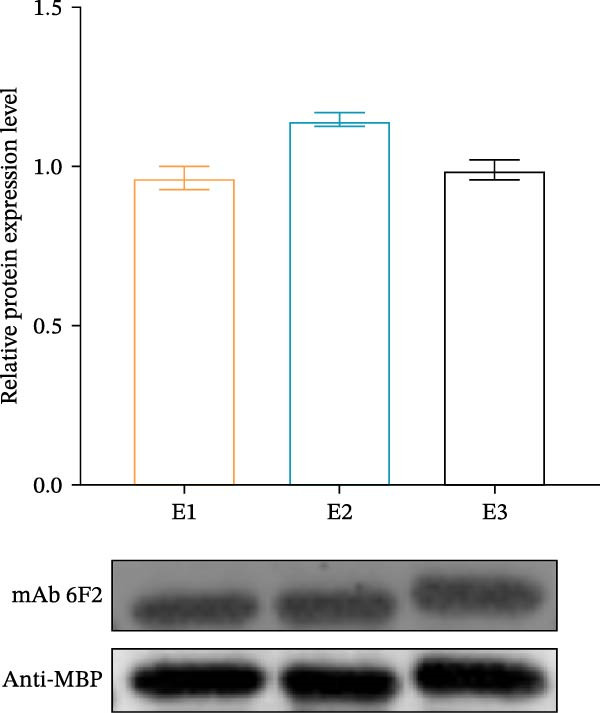
(D)

**Figure:**
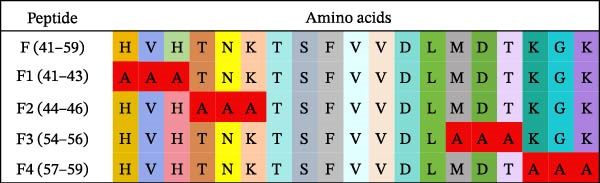
(E)

**Figure:**
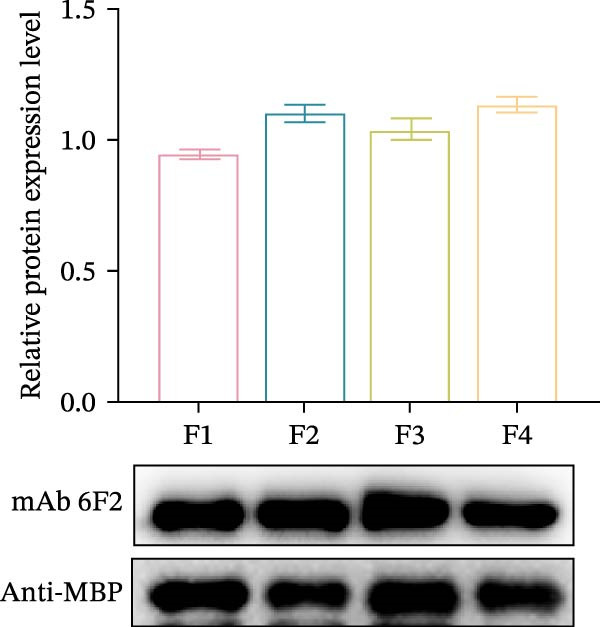
(F)

**Figure:**
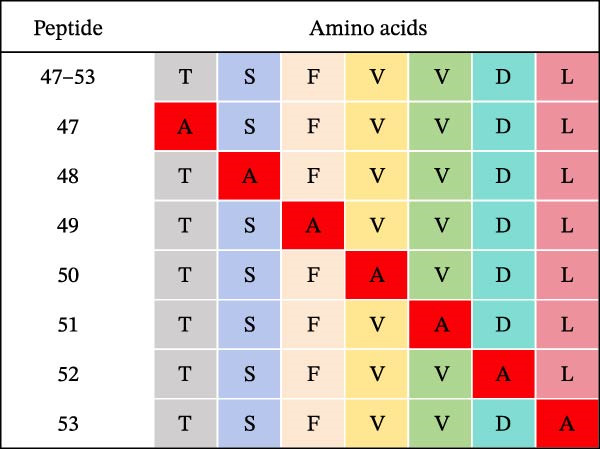
(G)

**Figure:**
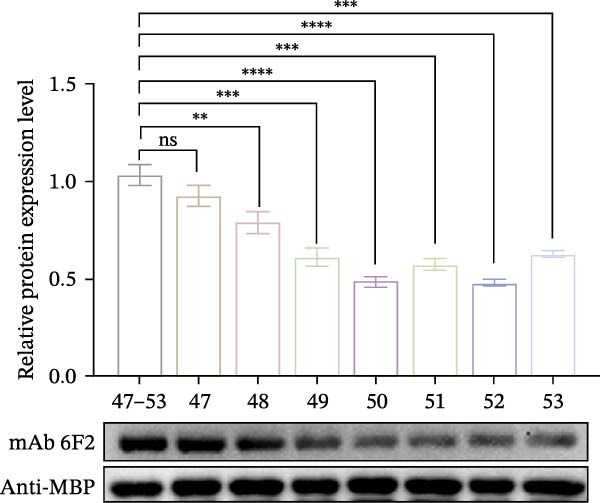
(H)

To further identify the critical amino acid residues within the linear epitope TSFVVDL, single‐alanine‐scanning mutagenesis was performed on each residue of this epitope, leading to the successful construction of seven independent mutants (Figure 4G). Western blotting analysis demonstrated that mutations of valine (V) at position 50 and aspartic acid (D) at position 52 within the TSFVVDL epitope significantly attenuated the binding capacity of monoclonal antibody (mAb) 6F2, indicating that residues V and D are critical for the recognition of this linear epitope by mAb 6F2. Meanwhile, quantitative densitometric analysis of the target protein via further validated this conclusion (Figure 4H).

Identification of 2F11 precise epitopes: We subdivided the epitopes (1–36 aa) identified by 2F11 into G1 (1–24 aa), G2 (10–30 aa), and G3 (13–36 aa) (Figure 5A). Western blotting results indicated that mAb 2F11 recognized G1 (Figure 5B), suggesting that the epitope lies within the residues 1–9. To identify the 2F11 epitope more accurately, alanine‐scanning mutagenesis with triple‐alanine substitutions was performed on residues 1–12 to construct four alanine mutants (Figure 5C). Western blotting analysis confirmed that mAb 2F11 failed to react with the mutant K3 (carrying alanine substitutions at 7–9 aa) (Figure 5D), indicating that the aa involved in K3 are necessary for the epitope. These results identified the core recognition sequence for mAb 2F11 binding to ^7^GAD^9^.

Figure 5Mapping of mAb 2F11 epitope in VP1. (A) Truncation strategy for VP1 residues 1–36 aa. Fragments G1 (1–24 aa), G2 (10–30 aa), and G3 (13–36 aa) were expressed. (B) Western blotting of G1–G3 probed with mAb 2F11. (C) Alanine‐scanning mutants (K1–K4) of 1–12 aa, the red area indicates a mutation. (D) Key residues were validated by Western blotting, with quantitative densitometric analysis performed. Data are presented as mean ± standard deviation (SD), and error bars represent the SD of three independent experiments. Statistical significance was defined as follows:  ^∗∗∗∗^ *p* < 0.0001.

**Figure:**
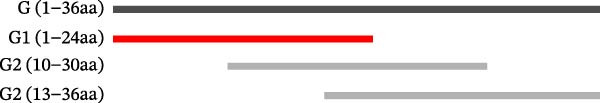
(A)

**Figure:**
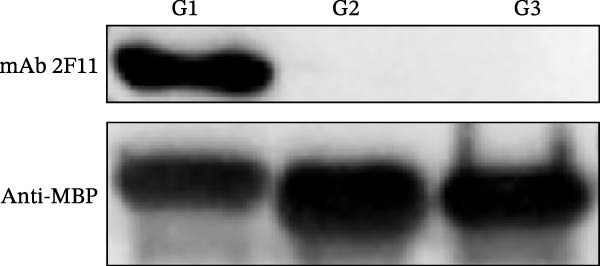
(B)

**Figure:**
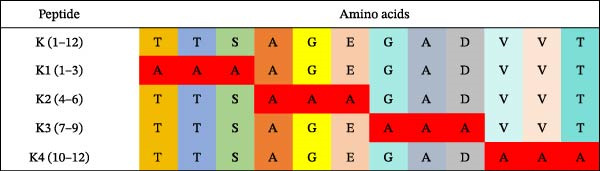
(C)

**Figure:**
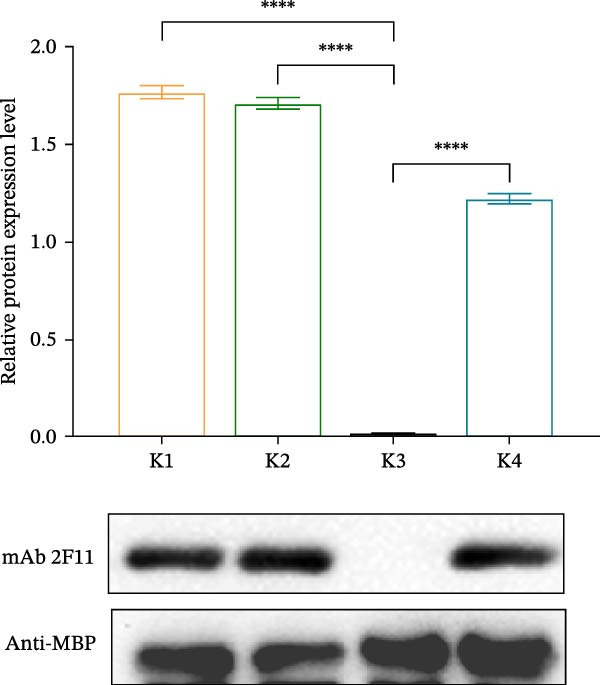
(D)

### 3.3. Homology Analysis of VP1 Epitope

To assess the conservation of the epitope region in different topotypes of the FMDV SAT serotypes, sequence alignment of VP1 from 53 SAT strains (35 SAT2, 13 SAT1, 5 SAT3) was performed. The core sequence GAD was 100% conserved across the majority of SAT2 topotypes, yet only 66.7% identical in one topotype VII strain (South Africa) and one topotype XII strain (Uganda). This core sequence also shared 100% identity with topotypes I, III, V, VII, and XII of SAT1 and topotypes II, III, and V of SAT3, while only 66.7% identity with other topotypes within SAT1 and SAT3 (Figure 6A). Collectively, ^7^GAD^9^ was highly conserved across all topotypes belonging to the SAT serotypes.

Figure 6Conservation analysis of VP1 antigenic epitopes. (A) Multiple sequence alignment of the ^7^GAD^9^ epitope recognized by mAb 2F11. (B) Multiple sequence alignment of the ^47^TSFVVDL^53^ epitope recognized by mAb 6F2. (C) Multiple sequence alignment of the ^204^RFDAPIGVE^212^ epitope recognized by mAb 3B2. (D) Multiple sequence alignment of the G–H loop.

**Figure:**
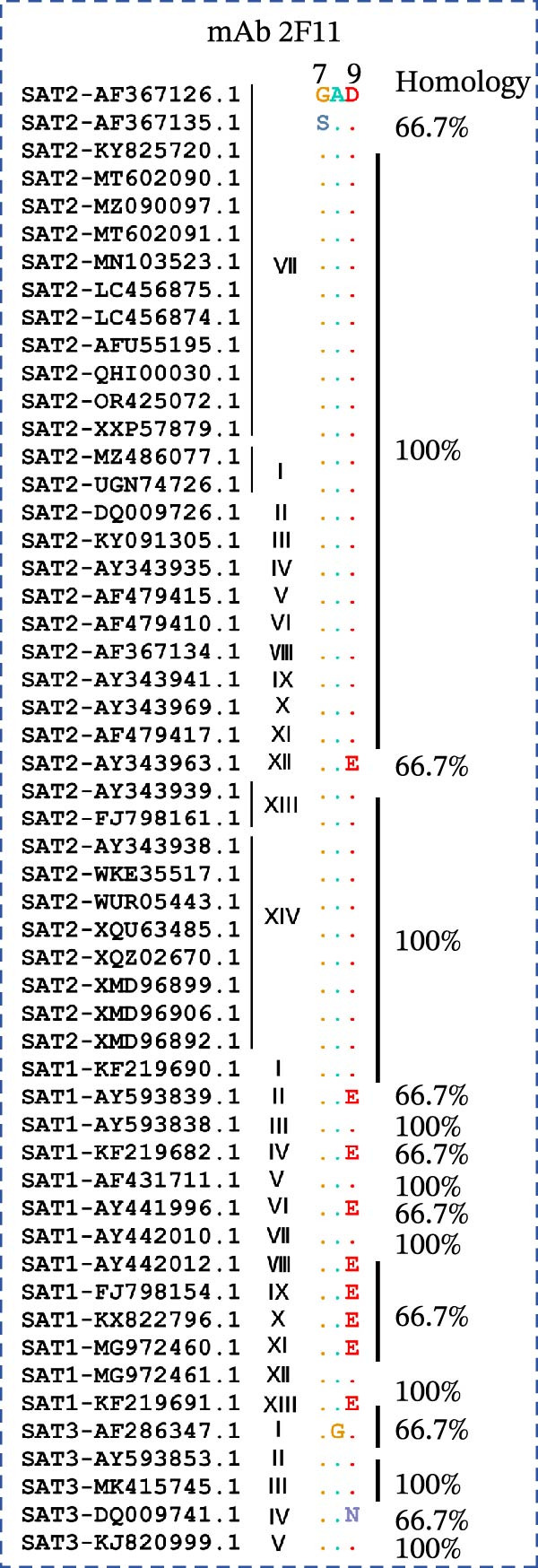
(A)

**Figure:**
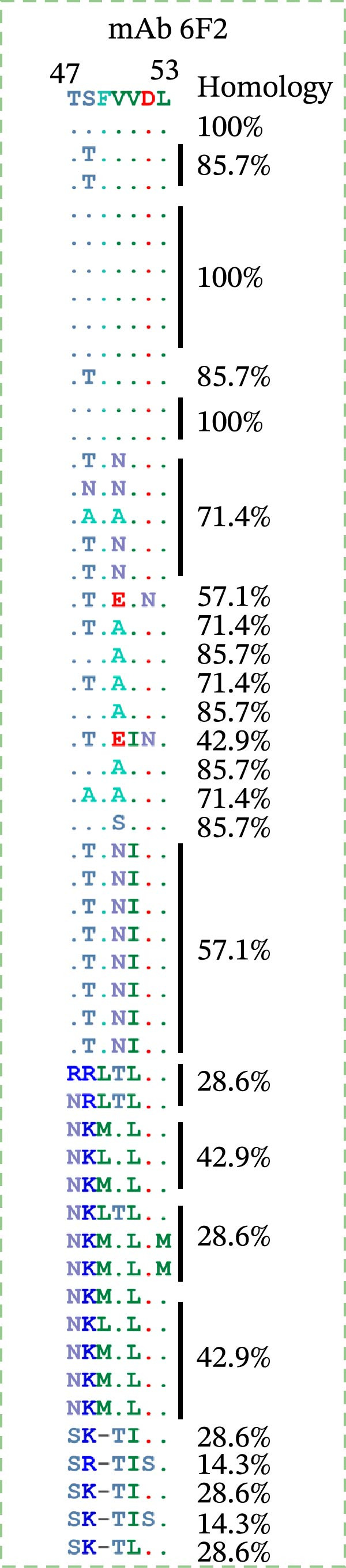
(B)

**Figure:**
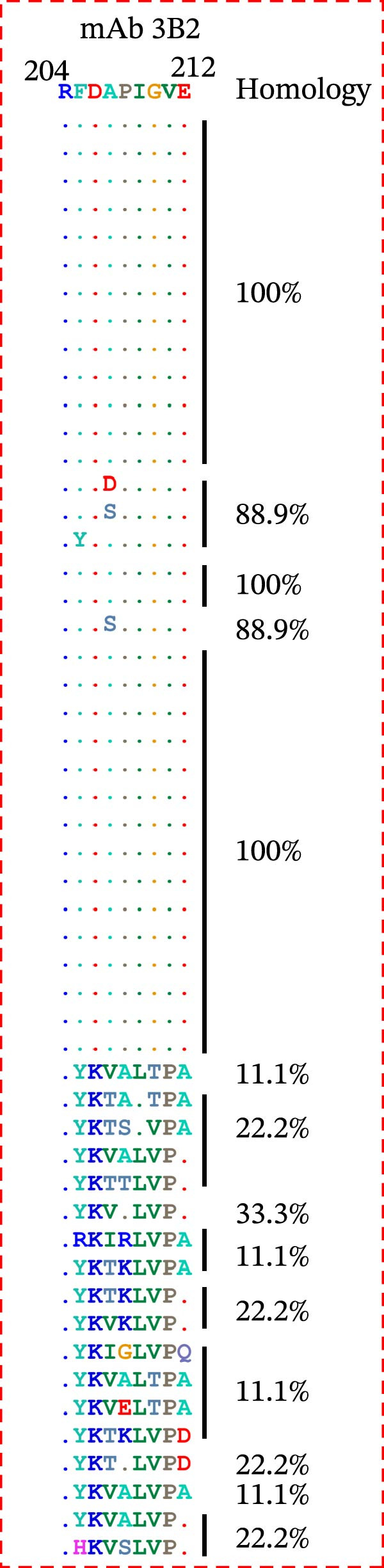
(C)

**Figure:**
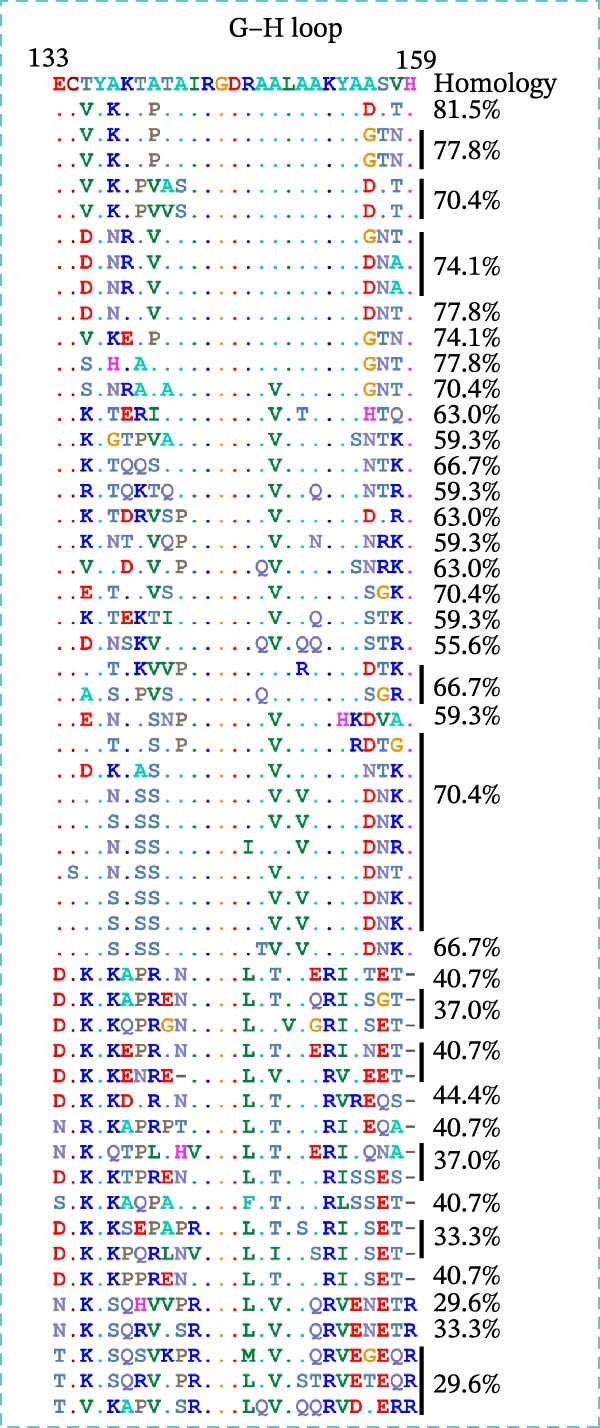
(D)

The epitope ^47^TSFVVDL^53^, recognized by mAb 6F2, was conserved exclusively within SAT2 topotype VII: specifically, it showed 85.7% sequence identity among strains isolated from Egypt, Ethiopia, and Sudan, while maintaining 100% identity in strains from other regions within this topotype. Beyond this, low conservation of TSFVVDL was observed with other SAT2 topotypes, as well as with SAT1 and SAT3 serotypes (Figure 6B).

Most importantly, the epitope ^204^RFDAPIGVE^212^ was highly conserved across all 14 SAT2 topotypes, exhibiting 88.9% identity in topotypes I, II, III, and VI and 100% identity in the remaining topotypes. In contrast, it showed minimal sequence similarity with SAT1 and SAT3 VP1 proteins, confirming its serotype specificity. The critical Asp206 residue was 100% conserved across all SAT2 topotypes (Figure 6C). By comparison, the G–H loop, a classical immunodominant region widely used in FMDV diagnostics, was shown via sequence alignment to exhibit substantial sequence variability among SAT2 topotypes (Figure 6D).

### 3.4. Structural Localization and Accessibility Analysis

**Figure 7:**
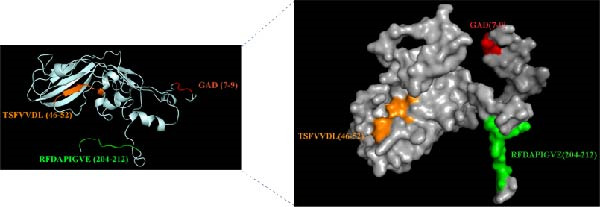
Structural localization and solvent accessibility of epitopes on SAT2 VP1. A three‐dimensional structural model of the SAT2 VP1 protein was generated using SWISS‐MODEL based on the crystal structure of FMDV. The spatial locations of the epitopes are highlighted: RFDAPIGVE (green), TSFVVDL (orange), and the GAD core motif (red). Both RFDAPIGVE and TSFVVDL are situated on the solvent‐exposed surface of the protein.

The three‐dimensional structure of the SAT2 VP1 protein was modeled using SWISS‐MODEL (https://swissmodel.expasy.org), and the spatial positions of the identified linear epitopes were visualized with PyMOL (Figure 7). Both the ^204^RFDAPIGVE^212^ (green) and ^47^TSFVVDL^53^ (orange) epitopes localize on the solvent‐exposed surface of VP1. Quantitative analysis of the SASA confirmed their high accessibility, with values of 922.6 Å^2^ and 1624.7 Å^2^ for ^204^RFDAPIGVE^212^ and ^47^TSFVVDL^53^, respectively. Although the key ^7^GAD^9^ motif (red) exhibited a lower SASA (219.4 Å^2^), its critical residues remained partially solvent‐accessible. This prominent surface localization and structural accessibility provide a direct structural basis for antibody recognition, supporting the potential of these epitopes to serve as targets in ELISA‐based serological detection.

## 4. Discussion

FMDV serotype SAT2, historically confined to sub‐Saharan Africa, has now emerged as a significant transboundary threat. Recent outbreaks in the Middle East (e.g., Iraq, Jordan, and Turkey during 2022–2023) and the escalating risks in East Asia, particularly in China’s swine industry, underscore its global spread [16, 26]. The presence of 14 genetically distinct SAT2 topotypes (Table 1) poses challenges for serological diagnosis due to minimal cross‐protection among them, emphasizing the inadequacy of current assays for comprehensive detection across various SAT2 topotypes [7, 27]. The lack of SAT2‐specific diagnostic tools in China underscores the critical need for highly sensitive and specific assays capable of detecting diverse SAT2 topotypes to enhance biosecurity readiness in high‐risk regions along international trade routes.

This study systematically identified linear epitopes within the immunogenic VP1 protein of SAT2. The epitope ^204^RFDAPIGVE^212^, recognized by mAb 3B2, was shown to be highly conserved across all 14 reported SAT2 topotypes (35 representative strains), exhibiting an overall sequence identity of 88.9% with only limited conservative amino acid substitutions. Notably, this epitope is located within a VP1 region corresponding to functionally important antigenic sites previously described in FMDV serotypes O and A [28]. The epitope exhibits broad conservation across all topotypes of FMDV serotype SAT2, making it a potential target for SAT2‐specific serodiagnosis. By contrast, the G–H loop, a classical immunodominant region widely used in FMDV diagnostics, displayed substantial sequence variability among SAT2 topotypes (Figure 6D), consistent with previous reports [27]. Such variability can significantly reduce the diagnostic sensitivity of G–H loop‐based assays when applied to genetically divergent field strains, particularly those belonging to emerging topotypes such as XIV [25]. In comparison, the RFDAPIGVE epitope exhibited markedly greater sequence stability across all analyzed SAT2 topotypes. This conserved nature directly addresses a key limitation of G–H loop–dependent assays and supports the potential utility of RFDAPIGVE as a more reliable diagnostic target. Furthermore, structural analysis revealed that RFDAPIGVE is surface‐exposed and characterized by high solvent accessibility (922.6), reinforcing its feasibility as an antigenic component for immunoassays.

The epitope ^47^TSFVVDL^53^ recognized by mAb 6F2 exhibited a highly restricted conservation profile: it was largely conserved within SAT2 topotype VII, yet showed sequence variation across non‐VII SAT2 topotypes as well as SAT1 and SAT3 serotypes, which limits its applicability for broad‐spectrum SAT2 diagnosis. The ^7^GAD^9^ motif, recognized by mAb 2F11, shows considerable conservation across the analyzed SAT serotypes (Figure 6A), suggesting it may represent an evolutionarily conserved core recognition sequence within the SAT serogroup. However, its broader diagnostic utility across all SAT serotypes necessitates further validation using larger panels of SAT1 and SAT3 field strains and sera. Future studies should incorporate recent epidemic strains to confirm the conservation of the core recognition sequence.

The diagnostic value of targeting conserved epitopes is further supported by recent studies on topotype‐specific immunoassays. For example, an ELISA developed using SAT2/XIV antigens demonstrated superior sensitivity for detecting homologous antibodies compared with assays based on SAT2/VII antigens [25], underscoring the profound impact of topotype‐level antigenic variation—largely driven by regions such as the G–H loop—on diagnostic performance. In this context, the identification of ^204^RFDAPIGVE^212^ as a conserved epitope across all SAT2 topotypes, including XIV, offers a promising solution. It provides a single, serotype‐specific molecular target that could underpin the development of a truly pan‐topotypic SAT2 diagnostic assay, thereby overcoming the topotype‐restricted sensitivity associated with current G–H loop‐based tests.

One limitation of this study should be acknowledged. The serotype specificity of mAbs 2F11 and 6F2 has not been fully validated against a comprehensive panel of FMDV serotypes. Future work will complete the serotype specificity profiling of these mAbs to fully characterize the antibody panel. In addition, further work should aim to expand the VP1 sequence analysis to include emerging SAT2 variants, particularly those from recent outbreak regions in West Asia, and to evaluate the diagnostic performance of the identified epitopes using larger panels of field sera. Ultimately, integrating multiple conserved epitopes, including ^204^RFDAPIGVE^212^, may facilitate the development of more robust SAT2 diagnostic platforms. These conserved antigenic regions may also offer valuable insights for the rational design of broadly protective SAT vaccines.

## 5. Conclusion

In this study, three linear epitopes within the VP1 protein of FMDV serotype SAT2 were systematically identified and characterized using specific mAbs. Among these, the epitope ^204^RFDAPIGVE^212^, recognized by mAb 3B2, showed strong conservation (88.9%–100%) across all 14 SAT2 topotypes analyzed. Based on in vitro epitope mapping and sequence analysis, this epitope represents a potential pan‐topotypic molecular target for SAT2‐specific serological diagnostics, though clinical validation with field sera is required to confirm its diagnostic sensitivity and specificity. In contrast, the ^47^TSFVVDL^53^ epitope, recognized by mAb 6F2, showed a more restricted conservation pattern, being largely specific to SAT2 topotype VII, which may limit its applicability for broad‐spectrum detection. The N‐terminal ^7^GAD^9^ motif, recognized by mAb 2F11, was identified as a core recognition sequence but displayed only partial conservation among SAT2 strains, suggesting limited diagnostic utility at the pan‐topotypic level. Collectively, these findings provide a rational basis for the development of improved SAT2‐specific diagnostic assays and contribute to a better understanding of antigenic conservation within this genetically diverse serotype.

## Author Contributions

All authors contributed to the research concept and design. Hong Tian, Hai‐xue Zheng, Yu‐qian Zhu, and Xin‐tai Shi conceived and designed the study. Yu‐qian Zhu, Xin‐tai Shi, and Alison Burman conducted the experiments. Yu‐qian Zhu, Xin‐tai Shi, Zheng‐wang Shi, Tao Xi, Huan‐cheng Liao, Jun‐cong Luo, Jie Chen, Lin Wang, Ya‐ge Xie, and Qian‐qian Yang analyzed the data. Yu‐qian Zhu and Xin‐tai Shi wrote the article. Zheng‐wang Shi and Donald P. King modified the article.

## Funding

This study was supported by the Key R&D Program of Gansu Province (Grant 26YFNA010) and Key Project of National Natural Sciences Foundation of China (Grant 32330107). Work at Pirbright is funded by the UK Department for Environment, Food and Rural Affairs (Project Number SE1131). The Pirbright Institute also receives grant‐aided support from the Biotechnology and Biological Sciences Research Council (BBSRC) of the United Kingdom (Project Numbers BBS/E/I/00007037, BB/X011038/1, BB/X011046/1, BBS/E/PI/230002C, and BBS/E/PI/23NB0004).

## Disclosure

All authors read and approved the final manuscript.

## Conflicts of Interest

The authors declare no conflicts of interest.

## Data Availability

The data that support the findings of this study are available from the corresponding author upon reasonable request.
